# The role of extracellular vesicles in circulating tumor cell-mediated distant metastasis

**DOI:** 10.1186/s12943-023-01909-5

**Published:** 2023-11-30

**Authors:** Siyin Guo, Jing Huang, Genpeng Li, Wenjie Chen, Zhihui Li, Jianyong Lei

**Affiliations:** Division of Thyroid Surgery, Department of General Surgery, West China Hospital, Sichuan University, Chengdu, Sichuan 610041 China

**Keywords:** Liquid biopsy, Extracellular vesicles, Circulating tumor cells, Tumor metastasis, Pre-metastatic niche, Biomarkers

## Abstract

Current research has demonstrated that extracellular vesicles (EVs) and circulating tumor cells (CTCs) are very closely related in the process of distant tumor metastasis. Primary tumors are shed and released into the bloodstream to form CTCs that are referred to as seeds to colonize and grow in soil-like distant target organs, while EVs of tumor and nontumor origin act as fertilizers in the process of tumor metastasis. There is no previous text that provides a comprehensive review of the role of EVs on CTCs during tumor metastasis. In this paper, we reviewed the mechanisms of EVs on CTCs during tumor metastasis, including the ability of EVs to enhance the shedding of CTCs, protect CTCs in circulation and determine the direction of CTC metastasis, thus affecting the distant metastasis of tumors.

## Introduction

Liquid biopsy is an emerging tumor diagnostic technique for tumor detection and monitoring that detects tumor-associated molecular markers in specimens by collecting body fluid samples for mainly analysis of circulating tumor DNA (ctDNA), CTCs and EVs [1, 2]. Among several markers of liquid biopsy, CTCs and EVs have good stability and detection sensitivity that can reflect the aggressiveness, metastatic potential, and prognosis of tumor cells [3–6]. In recent advances in liquid biopsy, EVs and CTCs have been commonly used as markers for liquid biopsies in tumor diagnosis [7], surveillance [8], prognosis [9], tumor classification and subtype discrimination [10]. CTC mRNA profiles are diverse and common, reflecting spatial tumor heterogeneity, and EV signals fluctuate greatly during treatment, thus reflecting temporal heterogeneity [11].

**Fig. 1:**
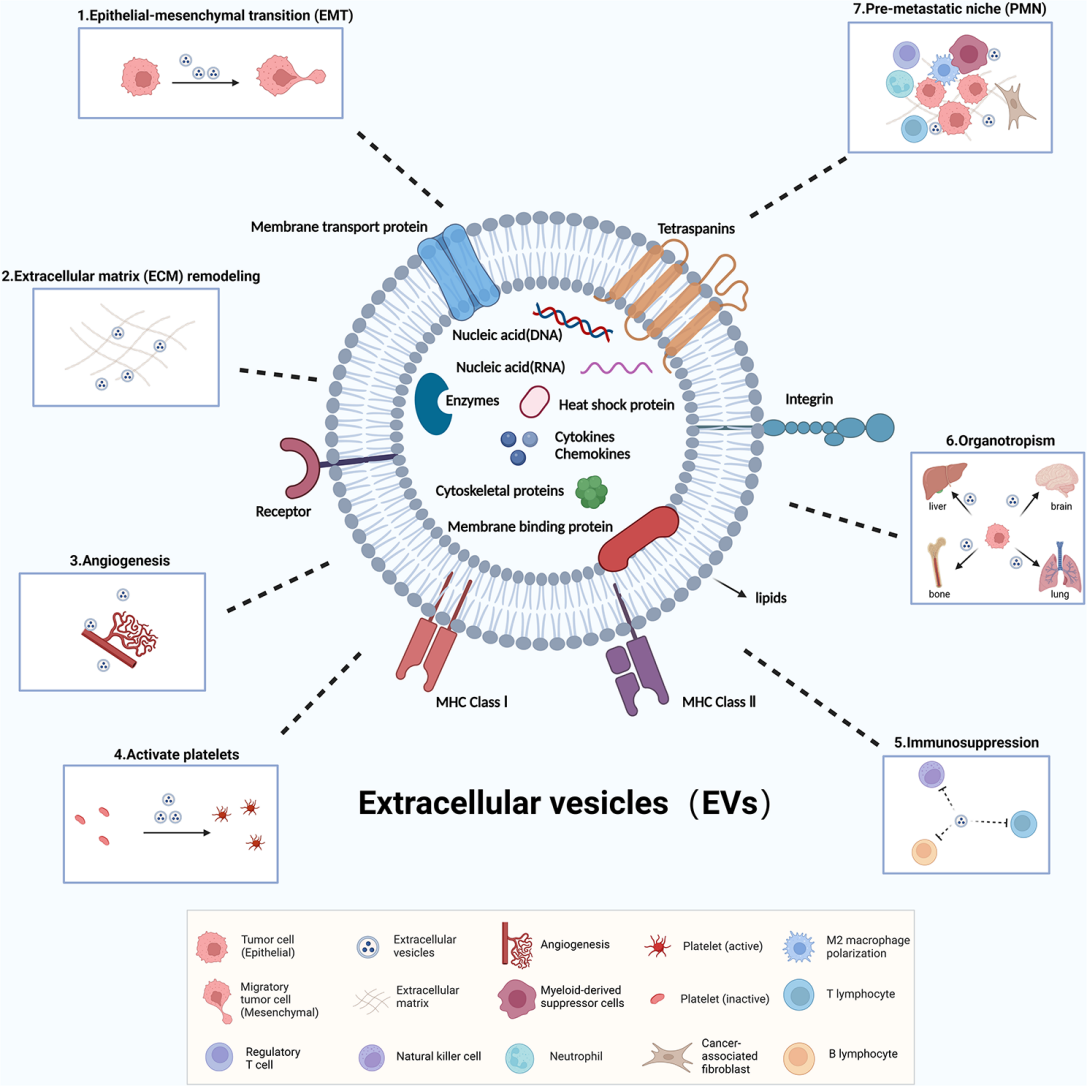
The composition of EVs and their role in tumor metastasis. The main substances of EVs are proteins, nucleic acids and lipids, which are the structural and functional basis of EVs. EVs play a role in key processes of tumor metastasis, such as promoting epithelial-mesenchymal transition (EMT), remodeling the extracellular matrix (ECM), promoting angiogenesis, activating platelets, immunosuppression, determining organotropism and promoting pre-metastatic niche (PMN) formation (Figure was created with BioRender.com).

EVs are a general term for particles that are naturally released by cells [12]. EVs are secreted via the endosomal pathway. First, the cell membrane is recessed inward to form early sorting endosomes. Then, late sorting endosomes are formed, and intraluminal vesicles (ILVs) are produced, further forming multivesicular vesicles (MVBs), which are partly degraded by lysosomes and partly released extracellularly to form EVs [13, 14]. According to the biogenesis pathway, EVs can be broadly classified into three categories: exosomes, microvesicles, and apoptotic bodies [15], which in this review we collectively refer to as EVs. In terms of structure, EVs contain proteins, nucleic acids and lipids **(**Fig. 1**)**, which play an important role in tumor formation, metastasis, and invasion [1, 14, 16–18]. Tumor-derived EVs in the blood are mainly secreted from primary tumor cells or released from burst CTCs [19]. Nontumor-derived EVs mainly refer to EVs from immune cells, platelets, and stem cells [20]. Here, we summarize tumor- and nontumor-derived EV cargoes that influence tumor progression **(**Table 1**)**.

**Table 1:** Tumor and nontumor derived EV cargoes affecting tumor progression

EV Source	Cargo	Pathway	Effect	Reference
CRC cell	miR-4299	Regulate target gene ZBTB4	Promote tumor proliferation and metastasis	Wu et al. (2023) [21]
	miR-224-5p	Downregulate CMTM4	Promote tumor progression	Wu et al. (2022) [22]
	miR-361-3p	Target TRAF3-mediated noncanonical NF-kB pathway	Promote tumor progression and proliferation	Li et al. (2021) [23]
	circPACRGL	Via miR-142-3p/miR-506-3p-TGF-β1 axis	Promote tumor progression	Shang et al. (2020) [24]
Gastric cancer cell	c-Myc	Via the KCNQ1OT1/miR-556-3p/CLIC1 axis	Promote tumor growth and metastasis	Li et al. (2022) [25]
	TGF-β	Via the TGF-β/Smad pathway	Promote tumor growth	Gu et al. (2012) [26]
HCC cell	lncRNA SNHG16	Via the miR-942-3p/MMP9 axis	Promote tumor metastasis	Xu et al. (2023) [27]
Lung cancer cell	PKM2	Via the AMPK pathway	Promote tumor progression	Zhou et al. (2022) [28]
Lung adenocarcinoma cell	circRAPGEF5	Via miR-1236-3p/ZEB1 axis	Promote the proliferation and metastasis of lung adenocarcinoma	Zhou et al. (2022) [29]
	miR-31-5p	Decrease SATB2 expression and increase MEK/ERK pathway activity	Promote tumor progression	Yu et al. (2021) [30]
TNBC cell	EphA2	Downregulate ZO-1 and activated the RhoA pathway in endothelial cells	Promote tumor metastasis	Liu et al. (2023) [31]
Renal cell carcinoma cell	lncRNA MALAT1	Regulate transcription factor ETS1 and affect TFCP2L1 activity	Promote the invasion and migration of renal cell carcinoma	Jin et al. (2021) [32]
Glioma cell	WT1	Via EV-mediated WT1-Thbs1 intercellular regulatory pathway	Promote tumor progression	Tsutsui et al. (2020) [33]
	miR-3184-3p	Promote M2 macrophage polarization	Promote tumor progression	Xu et al. (2022) [34]
	SBSN	Activate the NF-κB pathway	Promote tumor progression	Chen et al. (2022) [35]
Glioblastoma cell	VEGF-A	Interrupt the expression of claudin-5 and occludin	Promote tumor progression	Zhao et al. (2018) [36]
Pancreatic cancer cell	FGD5-AS1	Lead to M2 macrophage polarization	Promote tumor progression	He et al. (2022) [37]
	circPDK1	Sponge miR-628-3p to activate the BPTF/c-myc axis	Promote tumor progression	Lin et al. (2022) [38]
BC cell	miR-1910-3p	Target MTMR3 and activate the NF-κB signaling pathway	Promote tumor progression	Wang et al. (2020) [39]
Prostatic cancer cell	circFMN2	Via repression of KLF2/RNF128	Promote the proliferation, invasion and migration of prostatic cancer	Huang et al. (2023) [40]
Ovarian cancer cell	miR-106a-5p	Target KLF6	Promote the proliferation and metastasis of ovarian cancer	Zheng et al. (2022) [41]
	miR-205	Target VEGFA	Promote the proliferation, invasion and migration of ovarian cancer	Wang et al. (2019) [42]
Cervical cancer cell	miR-423-3p	Suppress macrophage M2 polarization	Inhibit the progression of cervical cancer	Yan et al. (2022) [43]
Endometrial cancer cell	LGALS3BP	Activate the PI3K/AKT/VEGFA signaling pathway	Promote the proliferation and migration of endometrial cancer	Song et al. (2021) [44]
CAFs^*^	miR-181b-3p	Regulate SNX2 expression	Promote the proliferation and migration of CRC	Jiang et al. (2022) [45]
	miR-625-3p	Inhibit the CELF2/WWOX pathway	Promote the progression of CRC	Zhang et al. (2022) [46]
	miR-1290	Target GSK3β	Promote the growth and metastasis of prostate cancer	Wang et al. (2022) [47]
	miR-18b	Regulate TCEAL7	Promote the invasion and metastasis of breast cancer	Yan et al. (2021) [48]
	circEIF3K	Via miR-214/PD-L1 axis	Promote the progression of CRC	Yang et al. (2021) [49]
	LncRNA LINC00659	Via miR-342-3p/ANXA2 axis	Promote the proliferation and migration of CRC	Zhou et al. (2021) [50]
MSCs^*^	miR-598	Target THBS2	Inhibit the proliferation and migration of NSCLC	Li et al. (2023) [51]
	miR-100	Via the miR-100/mTOR/miR-143 axis	Inhibit the progression of CRC	Jahangiri et al. (2022) [52]
	miR-744-5p	Inhibit M2 polarization of macrophages	Inhibit the progression of glioma	Liu et al. (2022) [53]
	miR-503-3p	Downregulate MEST	Inhibit the progression of human endometrial cancer	Pan et al. (2022) [54]
	miR-199a	Downregulate AGAP2	Inhibit the progression of glioma	Yu et al. (2019) [55]
	miR-1587	Downregulate the tumor-suppressor NCOR1	Promote the growth and proliferation of glioma	Figueroa et al. (2017) [56]
BMSCs^*^	LncRNA XIST	Via the miR-655/ACLY signal	Promote the growth and metastasis of osteosarcoma	Zhu et al. (2022) [57]
	NEAT1	Induce M2 macrophage polarization	Promote the progression of melanoma	Yang et al. (2022) [58]
	miR-342-3p	Inhibit the INHBA/IL13Rα2 axis	Inhibit the growth and metastasis of breast cancer	Liu et al. (2022) [59]
	miR-328-3p	Inhibit the Hippo pathway	Promote the progression of lung cancer	Liu et al. (2021) [60]
	miR-144	Downregulate CCNE1 and CCNE2	Inhibit the progression of NSCLC	Liang et al. (2020) [61]
	miR-205	Inhibit RHPN2	Inhibit the progression of prostate cancer	Jiang et al. (2019) [62]
TAM	miR-95	Bind to downstream target gene JunB	Promote the proliferation and invasion of prostate cancer	Guan et al. (2020) [63]
Platelets^*^	ITGβ3	Inhibit ferroptosis	Promote the metastasis of nasopharyngeal carcinoma	Li et al. (2022) [64]
	miR-24	Target mitochondrial mt-Nd2 and Snora75	Suppress solid tumor growth	Michael et al. (2017) [65]
	miR-223	Target tumor suppressor EPB41L3	Promote the invasion of lung cancer	Liang et al. (2015) [66]
NK cell^*^	miR-let-7b-5p	Target the cell cycle regulator CDK6	Inhibit the proliferation of pancreatic cancer cells	Di Pace et al. (2023) [67]
	miR-186	Induce inhibition of TGFβ signaling	Inhibit the growth of neuroblastoma	Neviani et al. (2019) [68]
CD8^+^ T^*^ cell	miR-765	Suppress PLP2 expression	Inhibit the progression of uterine corpus endometrial cancer (UCEC)	Zhou et al. (2021) [69]

CRC: colorectal cancer; HCC: hepatocellular carcinoma; NSCLC: non-small cell lung cancer; TNBC: triple-negative breast cancer; BC: breast cancer; CAFs: cancer-associated fibroblasts; MSCs: mesenchymal stem cells; BMSCs: bone marrow mesenchymal cells; TAM: tumor-associated macrophages; *: nontumor derived

**Fig. 2:**
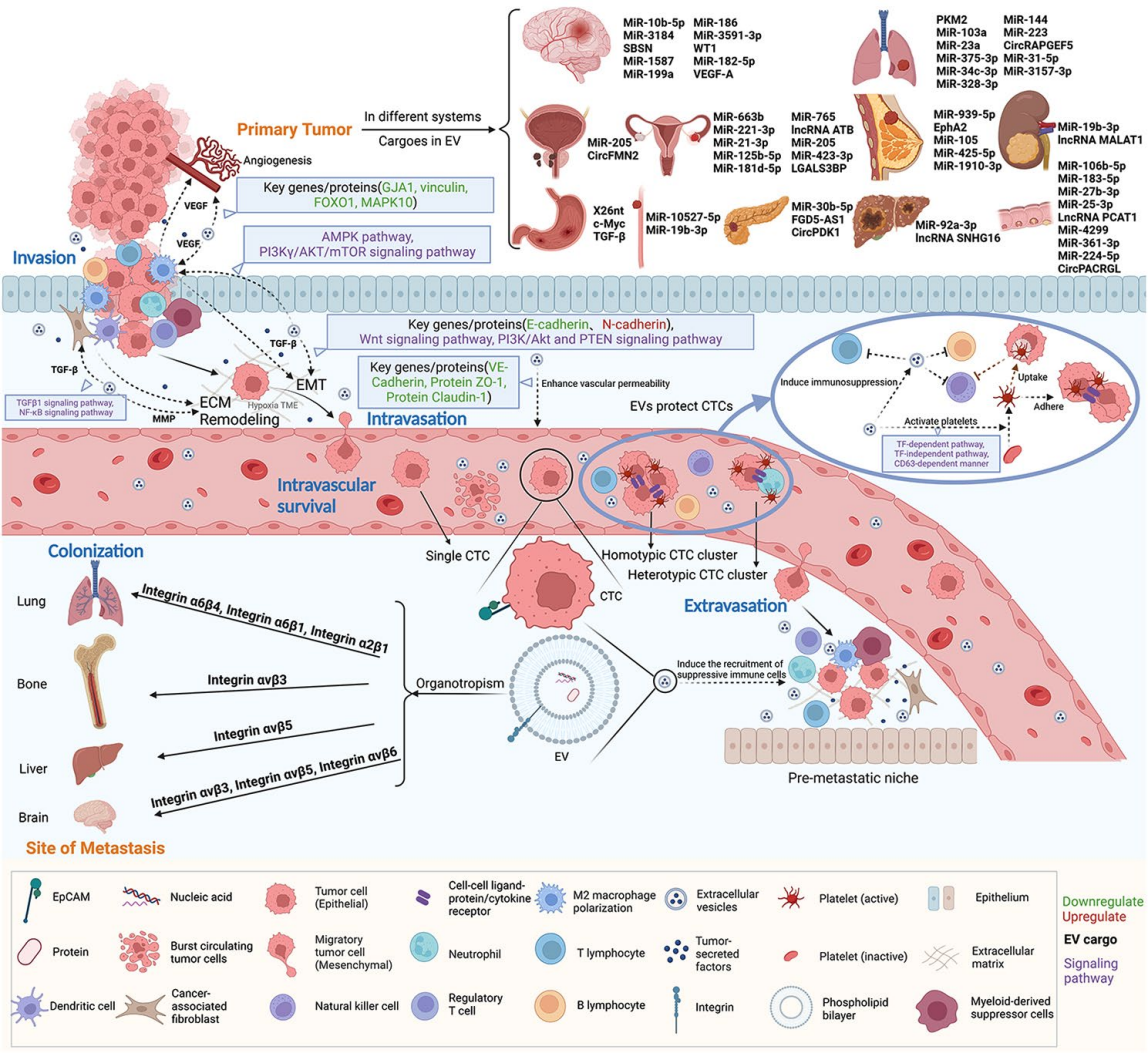
Role of EVs on CTCs during tumor metastasis. Tumor metastasis via the circulation involves several processes: invasion, intravasation, intravascular survival, extravasation, and secondary site colonization. At the beginning of metastasis, EVs enhance the shedding of CTCs by promoting epithelial-mesenchymal transition (EMT) and extracellular matrix (ECM) remodeling, as well as promoting angiogenesis and increasing vascular permeability; during metastasis, EVs protect CTCs by activating platelets and inducing immunosuppression; finally, EVs determine the metastatic direction of CTCs, participate in the formation of the pre-metastatic niche (PMN) and promote the metastasis and colonization of CTCs. The figure also summarizes the cargoes carried by EVs of different systemic tumor origins (Figure was created with BioRender.com).

CTCs are tumor cells that are shed from the primary tumor and enter the hematologic system with the potential to seed secondary tumors at newly metastatic sites [70]. CTC morphology may be different depending on the type, stage, and status of the tumor [71, 72]. CTCs can be released as a single cell or in homotypic or heterotypic clusters [73] **(**Fig. 2**)**. Cluster formation is produced in part by contact between a cell‒cell ligand‒protein or cytokine receptor and another cell [74]. An aggregate of more than one cancer cell is a homotypic CTC cluster; in combination with other cell types, such as immune cells or stromal cells, are heterotypic CTC clusters [75]. Compared to the single cells present in the circulation, these CTC clusters have an intercellular adhesion component that can promote tumor metastasis [76]. Data obtained from mouse models indicate that CTC clusters have a higher propensity to metastasize than CTCs [76]. With tumor growth, cancer cells, as single CTCs or CTC clusters, tend to break through the extracellular matrix (ECM) and infiltrate the circulation or transform into EpCAM CTCs, thereby reducing cell adhesion and promoting polarization and metastasis [77].

Tumor metastasis is important for disease progression and prognosis. The metastasis of tumors through circulation includes several stages: invasion, intravasation, intravascular survival, extravasation, and secondary site colonization **(**Fig. 2**)**. Studies [78–80] suggest that EVs protect CTCs during tumor metastasis. CTCs can be considered as seeds and the cargoes carried by EVs as fertilizer, where EVs can affect CTC colonization and growth in soil-like distant target organs. In other words, CTCs are a form of tumor cell presence that can reach distant organs and colonize through circulation, while various cellular sources of EVs can carry related molecules such as nucleic acids and proteins [81] that influence cell behavior and promote CTC-mediated metastasis.

This review summarizes the multiple mechanisms by which EVs regulate CTC-mediated distant tumor metastasis to better inform the early diagnosis, treatment, and prognosis of cancer.

## EVs enhance the shedding ability of CTCs

### EVs promote epithelial-mesenchymal transition (EMT) and extracellular matrix (ECM) remodeling

During the invasion phase, EMT leads to a decrease in tumor cell adhesion and can improve migration and invasion [82], facilitating tumor shedding to form CTCs. EVs play a vital role in EMT [83] and can regulate EMT through a variety of mechanisms. Specifically, EVs can promote EMT by carrying pro-EMT signaling factors, regulating key genes in the EMT pathway, activating the Wnt pathway and PTEN pathway, and promoting macrophage M2 polarization to enhance the shedding ability of CTCs **(**Table 2**)**.

EVs can carry many EMT-promoting factors, such as transforming growth factor-beta (TGF-β), which can activate the EMT pathway directly or indirectly [84, 85]. Moreover, EVs are rich in miRNAs that can regulate key genes in the EMT pathway through the downregulation of E-cadherin and upregulation of N-cadherin, thereby promoting EMT [86, 87]. Additionally, Fang et al. [88] reported that the long noncoding RNA (lncRNA) PCAT1 derived from colorectal cancer (CRC) EVs was found to regulate the activity of the Netrin-1-CD146 complex in CTCs, promoting EMT and liver metastasis in CRC. The Wnt pathway plays an important role in tumorigenesis and progression and can promote tumor progression through the activation of EMT [89]. Accumulating evidence [90] demonstrates that EVs are involved in the transfer of functional proteins and noncoding RNAs that trigger Wnt/β-catenin signaling to promote EMT. A more detailed study [91] has shown that EVs may also promote intercellular signaling of the Wnt pathway to prolong EMT in the tumor microenvironment (TME) [92]. PTEN overexpression promotes apoptosis and inhibits cell migration and invasion [93], and EVs can carry miRNAs to regulate EMT by affecting the PI3K/Akt and PTEN signaling pathways to promote CTC shedding [93, 94]. In addition to the mechanisms mentioned above, EVs can also promote EMT by promoting macrophage polarization to the M2 phenotype [95]. For example, according to a study [28], tumor-derived EV PKM2 induces M2 macrophage polarization via the AMPK pathway, leading to a decrease in E-cadherin and enhancement of N-cadherin and vimentin, thereby enhancing EMT in lung cancer. In another study [96], glioma cells selectively released tumor suppressive miR-3591-3p through EVs by targeting CBLB and activating the JAK2/PI3K/Akt/mTOR and STAT3 pathways to promote macrophage M2 polarization, and macrophages treated with miR-3591-3p mimics were enriched in TGFβ receptor signaling in EMT.

The TME includes immune cells, such as myeloid-derived suppressor cells (MDSCs), regulatory T cells (Tregs), tumor-associated macrophages (TAMs), dendritic cells, and B cells, as well as nonimmune cells and ECM, which play an important role in tumor recurrence and metastasis [97]. The ECM consists of proteins and carbohydrates that support basic cellular life activities, and diseases involving tumor invasion are attributed to the shift from homeostasis to remodeling of the ECM [98]. ECM is a prerequisite for tumor cell invasion and metastasis [99]. Within the hypoxic TME, EV levels increase and can mediate intercellular signaling [100–104]. EVs could enhance the remodeling of ECM and promote the invasion of tumor cells into the stroma, thereby promoting the detachment of CTCs from the primary site.

EVs can promote ECM remodeling directly by carrying ECM remodeling-related enzymes or indirectly by regulating stromal cells **(**Table 2**)**. A study [105] has shown that matrix metalloproteinases (MMPs) in EVs can degrade ECM proteins, leading to remodeling of the ECM. Fibroblasts are a major component of the tumor stroma and have been shown to play an important role in tumor progression [106]. EVs can trigger fibroblast differentiation into cancer-associated fibroblasts (CAFs) [107–110], thereby remodeling the ECM and facilitating the spread of tumor cells [99, 111, 112]. Mesenchymal stem cells (MSCs) are also important tumor stromal cells, and tumor cell-derived EVs activate pro-oncogenic pathways in MSCs and promote their conversion to CAFs [26], which is also beneficial for promoting ECM remodeling and thus facilitating CTC shedding.

### EVs promote angiogenesis and increase vascular permeability

Tumor-derived EVs can promote angiogenesis directly or indirectly by stimulating macrophages to release proangiogenic factors [113], which play an important role in the formation of CTCs from primary tumor shedding **(**Table 2**)**. In recent research [114], EV lncRNA ATB derived from ovarian cancer cells was shown to promote angiogenesis by regulating the miR-204-3p/TGFβR2 axis. A study [115] reported that hypoxic pancreatic ductal adenocarcinoma cell-derived EV miR-30b-5p promotes angiogenesis by inhibiting GJA1. Another study [116] found that EVs secreted by cervical cancer cells can deliver miR-663b to human umbilical vein endothelial cells (HUVECs) and inhibit the expression of adhesion protein (vinculin), thereby promoting angiogenesis and tumor growth. A previous study [117] found that EV miR-221-3p secreted by cervical cancer cells promotes angiogenesis by downregulating MAPK10 expression. EVs can also indirectly induce angiogenesis by stimulating VEGF release from macrophages. For example, lung cancer cell-derived EV miR-103a confers immunosuppressive and tumor-promoting phenotypes on macrophages, causing them to express high levels of the proangiogenic factors VEGF and angiopoietin-1, thereby promoting angiogenesis [118]. Recent studies have shown that tumor angiogenesis can be mediated not only by tumor cells but also by tumor stromal cells. For instance, Shi et al. [119] found that CAF-derived EVs can regulate CRC angiogenesis and progression by delivering VEGFA. CAF-derived EVs also upregulate miR-135b-5p, which promotes CRC cell growth and angiogenesis by inhibiting thioredoxin-interacting protein (TXNIP) [120].

Through endo-vascular penetration, tumor cells can be shed and enter circulation as CTCs [121]. Vascular barrier disruption is a critical step in CTC-mediated metastasis, which requires disruption of vascular tight junctions [122], and disruption of vascular endothelial cell integrity as well as consequent vascular permeability promotes subsequent metastasis of tumor cells [123], including the process of CTC extravasation [124]. Tumor-derived EVs can facilitate the production and metastasis of CTCs by transferring contents to endothelial cells to enhance vascular permeability **(**Table 2**)**. Mechanistically, EVs can be delivered into endothelial cells and then attenuate endothelial junction integrity by directly inhibiting the expression of vascular endothelial cadherin (VE-Cad), p120-catenin (p120) and zonula occludens-1 (ZO-1), which increase vascular permeability and promote tumor metastasis [125]. EV of EMT-CRC cell origin, miR-27b-3p, is transferred to endothelial cells and enhances vascular permeability by targeting VE-Cad and p120, which promote the production of CTCs [123]. A study [126] found that gastric cancer-derived EVs X26nt increase vascular permeability by directly binding VE-Cad in HUVECs. Researchers [127] found that breast cancer (BC)-derived EV miR-939-5p metastasizes from tumor cells to endothelial cells and directly targets VE-Cad, leading to disruption of tight junctions, thereby facilitating tumor cell entry into blood vessels to form CTCs by disrupting endothelial junctional integrity. In addition, EV miRNAs can target the tightly linked component protein ZO-1 [128]. A recent study [129] found that nasopharyngeal carcinoma-derived EV miR-455 increases vascular permeability by targeting ZO-1. Liu et al. [130] revealed that triple-negative breast cancer (TNBC)-derived EVs could transfer EphA2 protein to endothelial cells to enhance vascular permeability by downregulating ZO-1 and activating the RhoA pathway in endothelial cells, thus promoting tumor cell metastasis. In addition, EV-mediated miR-182-5p inhibits the tight junction-associated protein ZO-1, thereby enhancing vascular permeability and transendothelial migration of tumors [131]. A research project [132] reported that EV miR-25-3p in CRC promotes vascular permeability and tumor metastasis by targeting the ZO-1 protein. Hsu et al. indicated that hypoxic lung tumor cell-derived EV miR-23a upregulates the inhibition of the tight junction protein ZO-1 to increase vascular permeability [133], which suggests a potential association between EVs and CTCs. Moreover, a previous study [134] showed that EV-associated miR-105 in BC disrupts tight junctions by directly targeting the protein ZO-1. On the other hand, EV miR-375-3p could also promote small cell lung cancer metastasis by directly binding to the 3’UTR of the tight junction protein claudin-1 in vascular endothelial cells and negatively regulating its expression to disrupt the tight junctions in vascular endothelial cells [135]. Research [136] has shown that NSCLC-derived EV miR-3157-3p can translocate into vascular endothelial cells to target TIMP2/KLF2, promote angiogenesis and increase vascular permeability, thereby promoting tumor metastasis.

In addition to promoting angiogenesis, EVs can also promote tumor metastasis by promoting lymphangiogenesis. EVs circ_0026611 inhibit PROX1 acetylation and ubiquitination to promote lymphangiogenesis in esophageal squamous carcinoma [137]. EVs derived from gastric cancer cells deliver miR-1246 to lymphatic endothelial cells and promote lymphangiogenesis and lymphatic remodeling [138].

**Table 2:** Mechanisms by which EVs promote the shedding of tumor cells to form CTCs

Main Process	Theory	EV cargo	Mechanism	Type of effect	Reference
Epithelial-mesenchymal transition (EMT)	Carry the pro-EMT signaling factors	TGF-β1	Act as an early signal to induce the phosphorylation of SMAD2 in A549 cells to regulate EMT	Directly	Yin et al. (2020) [139]
	Regulate key genes in the EMT pathway	lncUCA1	Enhance EMT and activate metastasis through elevating Vimentin and MMP9 expression	Directly	Xue et al. (2017) [86]
		miR-19b-3p	Upregulate the expression of N-calmodulin, Vimentin, and Twist, and downregulate E-calmodulin to promote EMT	Directly	Wang et al. (2019) [87]
	Activate the Wnt pathway	miR-10527-5p	Affect EMT via Wnt/β-catenin signaling in vitro and in vivo	Directly	Xiao et al.(2023) [90]
	Activate the PTEN pathway	miR-19b-3p	Target the PTEN pathway to affect the expression of downstream EMT-related proteins	Directly	Zeng et al. (2020) [93]
		miR-92a-3p	Promote EMT progression by inhibiting PTEN and activating the Akt/snail signaling pathway	Directly	Yang et al. (2020) [94]
	Promote macrophage M2 polarization	PKM2	Induce M2 macrophage polarization via the AMPK pathway, thereby enhancing EMT	Indirectly	Zhou et al. (2022) [28]
		miR-3591-3p	Promote macrophage M2 polarization by targeting the CBLB and activating JAK2/PI3K/Akt/mTOR and STAT3 pathways, thus promoting EMT	Indirectly	Li et al. (2022) [96]
		miR-106b-5p	Promote macrophage polarization toward M2-like polarization by activating PI3Kγ/AKT/mTOR signaling pathway through downregulation of PDCD4 to activate macrophages promote EMT	Indirectly	Yang et al. (2021) [95]
Extracellular matrix (ECM) remodeling	Carry ECM remodeling-related enzymes	MMP	\	Directly	Tauro et al. (2013) [105]
	Trigger fibroblast differentiation into CAFs	miR-425-5p	Activate the TGFβ1 signaling pathway by suppressing TGFβRII expression, thereby promoting the conversion of human breast fibroblasts (HMF) to the CAF phenotype	Indirectly	Zhu et al. (2022) [107]
		lncRNA Gm26809	Reprogram fibroblasts into tumor-promoting CAFs through transfer of lncRNA Gm26809	Indirectly	Hu et al. (2019) [108]
		miR-630	Facilitate CAFs activation by inhibiting KLF6 and activating the NF-κB pathway	Indirectly	Cui et al. (2021) [109]
	Facilitate the conversion of MSCs to CAFs	TGF-β	Trigger the differentiation of hucMSCs to CAFs through EVs-mediated TGF-β transfer and TGF-β/Smad pathway activation	Indirectly	Gu et al. (2012) [26]
Angiogenesis	Promote angiogenesis	lncRNA ATB	Promote angiogenesis by regulating the miR-204-3p/TGFβR2 axis	Directly	Yuan et al. (2022) [114]
		miR-30b-5p	Promote angiogenesis by inhibiting GJA1	Directly	Chen et al. (2022) [115]
		miR-663b	Inhibit the expression of adhesion protein (vinculin), thereby promoting angiogenesis	Directly	You et al. (2021) [116]
		miR-183-5p	Promote angiogenesis through the regulation of FOXO1	Directly	Shang et al. (2020) [140]
		miR-221-3p	Promote angiogenesis by downregulating MAPK10 expression	Directly	Zhang et al. (2019) [117]
	Stimulate macrophages to release pro-angiogenic factors	miR-103a	Cause macrophages a high level of expression with pro-angiogenic factors VEGF and angiopoietin-1, thereby promoting angiogenesis	Indirectly	Hsu et al. (2018) [118]
Vascular permeability	Target vascular endothelial -Cadherin (VE-Cad)	miR-27b-3p	Enhance vascular permeability by targeting VE-Cad and p120	Directly	Dou et al. (2021) [123]
		X26nt	Increase vascular permeability by binding VE-Cad in HUVECs	Directly	Chen et al. (2021) [126]
		miR-939	Target VE-Cad and lead to disruption of tight junctions	Directly	Di Modica et al. (2017) [127]
	Target the tightly linked component protein zonula occludens-1(ZO-1)	miR-455	Increases vascular permeability by targeting ZO-1	Directly	Xie et al. (2023) [129]
		EphA2	Enhance vascular permeability by downregulating ZO-1 and activate the RhoA pathway in endothelial cells	Directly	Liu et al. (2022) [31]
		miR-182-5p	Inhibit the tight junction-associated protein ZO-1, thereby enhancing vascular permeability	Directly	Li et al. (2020) [131]
		miR-25-3p	Promote vascular permeability by targeting protein ZO-1	Directly	Zeng et al. (2018) [132]
		miR-23a	Upregulate inhibition of tight junction protein ZO-1 to increase vascular permeability	Directly	Hsu et al. (2017) [133]
		miR-105	Disrupt tight junctions by directly targeting protein ZO-1	Directly	Zhou et al. (2014) [134]
	Bind to the 3’UTR of the tight junction protein claudin-1	miR-375-3p	Bind to the 3’UTR of the tight junction protein claudin-1 in vascular endothelial cells and negatively regulate its expression to disrupt the tight junctions	Directly	Mao et al. (2021) [135]
	Target TIMP2/KLF2	miR-3157-3p	Promote angiogenesis and increase vascular permeability by targeting TIMP2/KLF2	Directly	Ma et al. (2021) [136]

## EVs promote tumor metastasis by protecting CTCs

### EVs activate platelets to protect CTCs

It has been shown that platelets can interact with tumor cells and influence tumor metastasis [141–143]. One mechanism is that tumor-derived EVs activate platelets to form tumor cell-induced platelet aggregates (TCIPA), and CTCs can become entangled in the thrombus formed by aggregated platelets [144], thus protecting CTCs from the deleterious effects of shear and giving them the advantage of evading immune surveillance by forming a physical barrier around them [145–148].

Specifically, tumor-derived EVs can directly activate platelets, thereby promoting thrombosis. Tumor cells express a procoagulant protein, tissue factor (TF), which can be released as TF-positive EVs [149, 150]. Previous studies [151, 152] have shown that TF on the surface of tumor-derived EVs is associated with platelet-activated aggregation and thrombosis and that EV-activated platelets can also be driven by TF-independent pathways, illustrating the complexity of the mechanism of tumor EV-induced platelet activation. For example, studies [152] have reported that BC-derived EVs induce platelet activation and aggregation through both independent and dependent mechanisms of TF, which may lead to cancer-associated thrombosis. Moreover, recent studies [153] have found that tumor-derived EVs deliver cancer markers in a CD63-dependent manner to activate platelets and promote thrombosis **(**Fig. 2**)**. Activated platelets form cell-fibrin-platelet aggregates around CTCs or stagnant tumor cells, providing mechanical protection and transferring MHC class I proteins to CTCs, interfering with the recognition of cancer cells by NK cells [154]. We predict that EVs may function in the activation of platelets to protect CTCs. Furthermore, recent study [155] showed that CTCs can uptake platelets, thus mediating immune escape, and more research is needed to explore whether this is also related to EVs.

Tumor-derived EVs not only protect CTCs by activating platelets to promote thrombosis but also enhance the adhesion of CTCs and promote metastasis. In a study [156], it was observed that EVs of hepatocellular carcinoma (HCC) origin induced ROS in HCC cells through SMAD3 signaling and regulated CTC adhesion, thereby promoting metastasis. In addition, during platelet activation or apoptosis, platelet extracellular vesicles can form [157], and they are associated with tumor progression [158]. Research [159] suggests that platelet-derived microparticles promote tumor cell invasiveness by stimulating the synthesis and secretion of MMP-2. Another study [160] showed that the characterization of the expression of promalignant genes and prothrombotic phenotypes in cancer cells by the crosstalk with platelet mEVs could provide prognostic information on cancer.

### EVs induce immunosuppression to protect CTCs from attack

While immune cells can recognize and attack CTCs under normal conditions, EVs can carry molecules that interact with circulating immune cells (T cells, NK cells, and B cells) to keep CTCs from attacking. Specifically, tumor-derived EVs carry immunosuppressive cargo, deliver molecular signals to immune cells, and participate in various immunosuppressive or immunostimulatory signaling pathways [161].

In particular, EVs mediate the immune escape of tumor cells by expressing programmed death ligand 1 (PD-L1) in the circulation [162]. PD-L1 is a type I transmembrane protein that binds to its receptor PD-1 to inhibit T-cell activation and thus maintain immune homeostasis [163]. PD-L1 can be expressed on tumor-derived EVs and induce immunosuppression by binding to PD-1 on T cells [164–166], and PD-L1 on EVs is significantly upregulated in patients with distant tumor metastasis [167, 168], which indicates that EVs may potentially mediate the immune escape of CTCs. Based on this principle, anti-PD-1/PD-L1 immune checkpoint therapies have recently blocked PD-1/PD-L1 binding, allowing T cells to work and inhibiting tumor growth [163]. Referring to some existing studies [169, 170], we consider that targeting the secretion of EVs may be a way to counteract EVs. In addition, research has found that PD-L1 can also be detected on the surface of CTCs in many cancer types [171]. Moreover, EVs with FasL expression are able to cause apoptosis of antitumor CD8^+^ T cells [172], probably due to the interaction between MHC class I of EVs and CD8^+^ receptors of T cells, which leads to apoptosis of T cells by activating the Fas/FasL signaling pathway [161, 173], thereby producing an immunosuppressive effect.

NK cells are a subset of lymphocytes that can participate in antitumor immune responses [174]. In terms of EVs helping CTCs evade immune surveillance, NKG2D plays an important role in the cytotoxicity of NK cells [175], and EVs expressing ligands of NKG2D lead to impaired toxic function of NK cells [176], which may also mediate immune escape of CTCs. The BCR pathway controls B-cell survival, proliferation and activation and is a key player in the B-cell signaling mechanism [177]. A recent study [178] suggests that EVs may inhibit B-cell proliferation and survival through the expression levels of coreceptors involved in the negative regulation of BCR signaling.

## EVs determine the direction of metastasis and colonization of CTCs

During the whole transfer process, each type of cancer has a specific metastatic pathway, and cancer metastasis usually follows the target distribution of CTCs to preferred organs, which is called “organotropism” or “organ-specific metastasis“ [179]. EVs can provide metastatic organotropism, meaning that EVs can help determine the direction of CTC metastasis and promote CTC distant metastasis and colonization by participating in the formation of a pre-metastatic niche (PMN).

### Tumor-derived EV integrins determine organotropism

The amount of specific integrins in EVs seems to be responsible for the increased expression of the S100 gene in target cells and ultimately for the effect on organs [121]. Hoshino et al. [180] showed that EV proteomics reveals different integrin expression patterns and fusion to target cells in a tissue-specific manner to direct organ-specific colonization with EV integrins α6β4 and α6β1 associated with lung metastasis and EV integrin αvβ5 associated with liver metastasis. EVs expressing integrins α6β1 and α6β4 are directed to lung epithelial cells and lung fibroblasts, and integrin α6β4 also promotes lung metastasis by promoting S100 gene expression and the Src signaling pathway; EVs expressing integrin αvβ5 promote liver metastasis by binding to resident macrophages (Kupffer cells) in the liver and upregulating cell migration genes and S100 production genes [180, 181]. In addition, Huang et al. [182] found that EVs with low levels of miR-34c-3p can accelerate non-small cell lung cancer (NSCLC) invasion and migration by upregulating integrin α2β1. In a further study [183], tumor-derived αvβ3 integrin was associated with prostate cancer bone metastasis. Moreover, αv integrins are highly expressed in brain metastases [184, 185], where αvβ3, αvβ5 and αvβ6 integrins are associated with lung cancer brain metastases [186] **(**Fig. 2**)**.

### EVs are involved in the formation of the pre-metastatic niche (PMN)

The primary tumor establishes a microenvironment conducive to tumor growth through tumor-derived factors before CTCs reach the predetermined secondary sites of metastatic disease, referred to as the PMN [187, 188]. The marker of PMN formation is the recruitment of immune cells to establish an immunosuppressive microenvironment [189].

Tumor-derived EVs induce the recruitment of suppressive immune cells such as TAMs, tumor-associated neutrophils, Tregs, and MDSCs to distant secondary sites, thereby suppressing the antitumor immune response [190], promoting PMN formation and facilitating the colonization of CTCs. Tregs are the primary immunosuppressive cells in tumor immunity. They efficiently infiltrate and adapt to the TME and suppress antitumor immune responses, and inhibition of Treg infiltration in the TME increases tumor immune responses [191]. A study [192] found that tumor-derived EVs play a key role in the induction of Treg infiltration into pulmonary PMN by upregulating fibroblast-derived CCL1 production. Recently, a study [193] also revealed a novel mechanism of LC3-EV-enhanced lung PMN formation.

EVs can also participate in PMN formation by carrying loadings that affect the phenotype or function of the recipient cells. Specifically, tumor-derived EVs produced in the hypoxic microenvironment of tumor cells can polarize macrophages to the M2 phenotype in a HIF-1α- or HIF-2α-dependent manner, which facilitates the establishment of an immunosuppressive microenvironment, thus contributing to PMN formation [194, 195]. It was recently found that under hypoxic conditions, epithelial ovarian cancer cell-derived EVs deliver miR-21-3p, miR-125b-5p, and miR-181d-5p to macrophages and induce polarization of M2 macrophages, promoting tumor cell proliferation and migration [196]. Hypoxic glioma-derived EVs deliver miR-10b-5p through the NEDD2L/PIK4CA/PI3K/AKT axis to accelerate macrophage M2 polarization and promote glioma progression [197]. Moreover, EVs can affect receptor cell function, and dormant cancer cell-derived EVs promote PMN formation and cancer cell survival in the bone marrow by reprogramming the metabolic processes of bone marrow mesenchymal cells through the transfer of EVs IGF-2 and IGFBP2 [198].

### EVs promote distant tumor metastasis

**Fig. 3:**
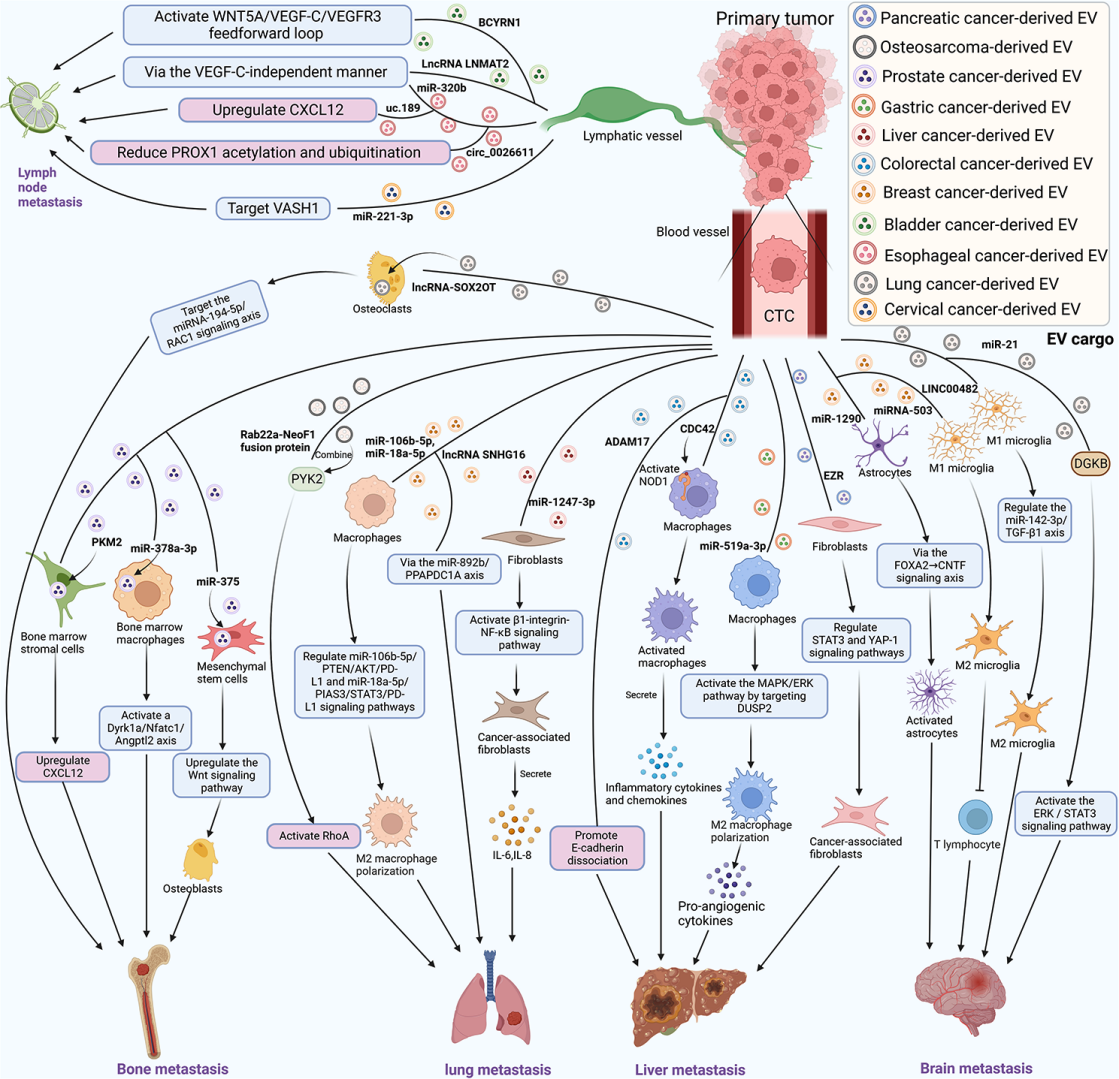
EVs determine CTC colonization in different distant metastatic sites. EVs of various tumor origins promote the metastasis of CTCs to bone, lung, liver and brain, and the mechanisms by which EVs promote lymph node metastasis are also illustrated in the figure (Figure was created with BioRender.com).

Organotropic metastasis is the tendency of certain primary tumors to spawn and dominate secondary tumors at distant metastatic sites in specific organs [199]. Additionally, EVs can promote lymphangiogenesis [137, 200] and lymph node metastasis of primary tumor cells [201–207]. We next summarize some of the mechanisms by which EVs determine CTCs colonization in different distant metastatic sites **(**Fig. 3**)**. Prostate cancer is most likely to develop bone metastases, and prostate cancer-derived miR-378a-3p-containing EVs promote osteolysis by activating the Dyrk1a/Nfatc1/Angptl2 axis in bone marrow macrophages during tumor bone metastasis [208]. Another study [209] found that primary prostate cancer cells educate the bone marrow to promote bone metastasis through primary prostate cancer EV-mediated transfer of PKM2 into bone marrow mesenchymal cells and subsequent upregulation of CXCL12. Tumor-derived EV miR-375 directly targets DIP2C and upregulates the Wnt signaling pathway to promote prostate cancer bone metastasis [210]. In another study [211], EV lncRNA-SOX2OT promotes bone metastasis of NSCLC by targeting the miRNA-194-5p/RAC1 signaling axis in osteoclasts. Bone metastases are also the preferred site of metastasis for BC. EVs with high CDH11 and ITGA5 expression (CDH11^high^/ITGA5^high^ EVs) produced by BC cells contribute to the formation of an osteogenic PMN in bone, further facilitating RUNX2^high^ cancer cell colonization and metastasis to bone [212].

In brain metastases from BC, BC-derived EV miR-1290 activates astrocytes in the brain metastasis microenvironment via the FOXA2→CNTF signaling axis and promotes tumor brain metastasis [213]. In another study [214], the loss of X-inactive-specific transcript (XIST) augment the secretion of EV miRNA-503, which promote brain metastasis in BC by affecting tumor cells and the tumor microenvironment. Furthermore, EV lnc-MMP2-2 upregulates EPB41L5 expression by sponging miR-1207-5p, and then EPB41L5 directly promotes endothelial-to-mesenchymal transition and destroys tight junctions, which ultimately promotes brain metastasis in NSCLC [215]. Another study [216] also reported that EVs transmit LINC00482 to regulate the miR-142-3p/TGF-β1 axis, induce microglial M2 polarization and affect the PMN, thus enhancing brain metastasis of NSCLC. Tiong et al. [217] reported that lung cancer-derived EVs expressing miR-21 promote lung cancer brain metastasis by inhibiting DGKB activation of the ERK/STAT3 signaling pathway.

The lung is rich in blood supply and is a metastatic site for many tumors. For instance, EVs carrying miR-200 promote BC cell colonization in the lung [218]. BC cells transmit sEV-miR-106b-5p and sEV-miR-18a-5p to macrophages and induce PD-L1 expression through the PTEN/AKT and PIAS3/STAT3 signaling pathways, which also leads to macrophage polarization and the development of BC lung metastasis [219]. Xia et al. [220] found that BC-derived EVs promote BC lung metastasis by carrying the lncRNA SNHG16 through the miR-892b/PPAPDC1A axis. In another study [221], tumor-derived EVs carrying miR-1247-3p promote lung metastasis of HCC by downregulating B4GALT3 to convert fibroblasts into CAFs and activating the β1-integrin-NF-κB signaling pathway. In a mouse experiment [222], EVs were found to carry the Rab22a-NeoF1 fusion protein to promote osteosarcoma cell metastasis to the lung via PYK2 activation of RhoA in donor cells in osteosarcoma. Furthermore, recent studies [193] have demonstrated new mechanisms by which EVs promote lung metastasis: tumor-derived LC3 EVs activate lung fibroblasts to produce CCL2 via the HSP60-TLR2‐MyD88‐NF‐κB pathway, which can recruit Mos to the lungs, contribute to the formation of pulmonary PMN and ultimately promote metastasis.

EVs also play an important role in CTC-mediated liver metastasis of CRC. Tumor-derived EV miR-934 can promote CRC liver metastasis by regulating the crosstalk between CRC cells and TAMs [223]. Wei et al. [224] found that CRC-EVs with a high cargo of CDC42 could activate NOD1 to promote tumor liver metastasis. CRC-derived EV miR‐181a‐5p activates hepatic stellate cells (HSCs) via regulation of IL6/STAT3 signaling, which promotes secretion of CCL20 from α‐HSCs and further activation of the ERK1/2/Elk‐1 pathway via CCR6 and upregulation of miR‐181a‐5p in CRC cells, ultimately resulting in liver metastases [225]. CRC-derived EVs carrying ADAM17 promote E-cadherin cleavage and enhance the migratory properties of CRC cells, which in turn facilitate liver metastasis in vivo [226]. Additional mechanisms involve the formation of an immunosuppressive microenvironment. CRC-derived EVs carrying TGF-β1 induce the formation of immunosuppressive ecological sites in the liver prior to metastasis and promote tumor metastasis [227]. Furthermore, recent studies [228] have shown that hepatocyte-derived EVs in fatty liver enhance the progression of CRC liver metastases by promoting oncogenic Yes-associated protein signaling and an immunosuppressive microenvironment. Recently, Chang et al. [229] found that pancreatic cancer-derived EV-EZR regulates the STAT3 and YAP-1 signaling pathways and promotes fibroblast activation for pancreatic cancer liver metastasis. In addition, another study [230] found that gastric cancer-derived EV miR-519a-3p activates the MAPK/ERK pathway by targeting DUSP2, which leads to M2-like polarization of macrophages, ultimately leading to gastric cancer liver metastasis.

## Conclusions and perspectives

This paper reviews the mechanisms by which EVs affect CTCs during their detachment from the primary tumor, their survival in the circulation and eventual colonization to distant sites in recent years. The process is complex, and although a large number of mechanisms have been described, many still remain to be researched. Learning more about these mechanisms can inspire us to gain a deeper understanding of tumor metastasis and facilitate our thinking about tumor prevention, diagnosis, and therapeutic strategies.

We conclude that EVs promote CTC-mediated metastasis and progression through a variety of mechanisms, including (i) EVs promote EMT by carrying pro-EMT signaling factors, regulating key genes and pathways in EMT, and promoting macrophage M2 polarization; EVs promote ECM remodeling by carrying ECM remodeling-related enzymes and regulating stromal cells; and EVs promote angiogenesis by directly or indirectly releasing proangiogenic factors and promote vascular permeability by targeting proteins, such as VE-Cad and ZO-1, which enhance the detachment of CTCs; (ii) EVs protect CTCs by activating platelets to form thrombus, and by carrying molecules that interact with circulating immune cells such as T cells, NK cells and B cells in the circulatory system; and (iii) EVs determine tumor organotropism by carrying integrins, as well as carrying cargoes such as proteins and nucleic acids to regulate relevant pathways and thus participate in PMN formation, ultimately facilitating the colonization of CTCs at distant sites.

By reading the relevant literature, we also found that there are still some mechanisms to be investigated for the role of EVs on CTCs. In circulation, CTCs can internalize platelets to express specific antigens to bind to NK cells, but the exact mechanism by which CTCs uptake platelets is currently unknown. It may be that EVs from the primary tumor domesticate platelets and thus lead to uptake, which is a direction that needs to be researched in the future. Whether the binding of CTCs to immune cells is a direct contact or EVs from primary tumor sources are first released into the bloodstream to interact with immune cells before inducing immunosuppression is a process that also needs to be investigated. In addition, there are many mechanisms involved in the process of EV-induced immunosuppression in tumors, but which one plays the dominant role and its specific causes need to be further investigated. Furthermore, there are more EV integrins that determine the shedding of CTCs from the primary tumor to specific distant metastatic sites that need to be discovered, and it is also necessary to think about whether there are other mechanisms of EV origin influencing organotropism in addition to EV integrins, and all these need to be researched further. We believe that further research on the role of EVs on CTCs during metastasis will also help to identify new biomarkers in liquid biopsies, which will contribute to the development of more innovative and precise oncology therapies to provide greater assistance to cancer patients.

## Data Availability

Not applicable.

## Abbreviations

EVs: Extracellular vesicles
CTCs: Circulating tumor cells
EMT: Epithelial-mesenchymal transition
ECM: Extracellular matrix
PMN: Pre-metastatic niche
CRC: Colorectal cancer
HCC: Hepatocellular carcinoma
NSCLC: Non-small cell lung cancer
TNBC: Triple-negative breast cancer
CAFs: Cancer-associated fibroblasts
MSCs: Mesenchymal stem cells
BMSCs: Bone marrow mesenchymal cells
TGF-β: Transforming growth factor-beta
miRNA: MicroRNA
lncRNA: Long noncoding RNA
TME: Tumor microenvironment
MDSCs: Myeloid-derived suppressor cells
Tregs: Regulatory T cells
TAM: Tumor-associated macrophages
MMP: Matrix metalloproteinase
HUVECs: Human umbilical vein endothelial cells
VE-Cad: Vascular endothelial cadherin
ZO-1: Zonula occludens-1
TF: Tissue factor
BC: Breast cancer
PD-L1: Programmed death ligand 1
